# Termitomenins F and G, Two New Lignan Glucosides from *Terminalia chebula* var. *tomentella* (Kurz) C. B. Clarke

**DOI:** 10.1007/s13659-021-00314-z

**Published:** 2021-06-10

**Authors:** Jun Yin, Hong-Tao Zhu, Man Zhang, Dong Wang, Chong-Ren Yang, Ying-Jun Zhang

**Affiliations:** 1 State Key Laboratory of Phytochemistry and Plant Resources in West China, Kunming Institute of Botany, Chinese Academy of Sciences, Kunming, 650201 People’s Republic of China; 2 University of Chinese Academy of Sciences, Beijing, 100049 People’s Republic of China; 3 Yunnan Key Laboratory of Natural Medicinal Chemistry, Kunming Institute of Botany, Chinese Academy of Sciences, Kunming, 650201 People’s Republic of China

**Keywords:** *Terminalia chebula* var. *tomentella*, Lignan glucosides, Hydrolyzable tannins, α-Glucosidase inhibitory activity, Calculated ECD

## Abstract

The extensive chemical investigation on the branches and leaves of *Terminalia chebula* var. *tomentella* (Combretaceae) led to the isolation of two new lignan glucosides with a furofuran skeleton, termitomenins F (**1**) and G (**2**). In addition, 19 known compounds including five lignan glucosides (**3**–**7**), six hydrolyzable tannins (**8**–**13**) and eight simple phenolics (**14**–**21**) were also identified. Their structures were determined by comprehensive spectroscopic analyses. It is noted that **8** and **9** were *C*-glycosidic hydrolyzable tannins with one hexahydroxydiphenoyl and one gallagyl group linked to an open-chain glucosyl *C*-1/*O*-2/*O*-3 and *O*-4/*O*-6, respectively, which were rarely found in plants. Nine known compounds, **6**–**9**, **13**, and **18**–**21**, were procured from the titled plant for the first time, while **3**–**5**, **10**–**12** and **14**–**17** were also found in the fruits. Notably, the known hydrolyzable tannins **8**–**13** exhibited stronger α-glucosidase inhibitory activities with IC_50_ values ranging from 0.10 to 3.12 μM, than the positive control, quercetin (IC_50_ = 9.38 ± 0.33 μM).

**Supplementary Information:**

The online version contains supplementary material available at 10.1007/s13659-021-00314-z.

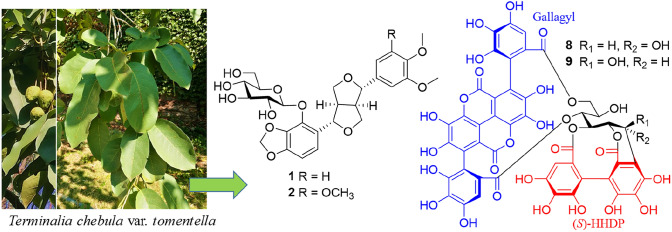

## Introduction

*Terminalia* Linn, the second largest genus in the family Combretaceae, is distributed globally in the tropical and subtropical areas. Among which, some species, such as *T. catappa* Linn, *T. bellirica* Roxb, and *T. chebula* Retz, are widely used medicinal plants. Particularly, *T. chebula* is a famous and commonly used medicinal plant in Ayurveda, Tibetan, and traditional Chinese medicinal systems. So far, 39 *Terminalia* species have been chemically and pharmacologically studied, from which 368 compounds, including terpenoids, hydrolyzable tannins, flavonoids, lignans, phenols and glycosides with a wide range of bioactivities, e.g., liver and kidney protection, antibacterial, anti-inflammatory, anticancer, immune regulation, anti-diabetes, and wound healing, were reported [1].

*Terminalia chebula* var. *tomentella* (Kurz) C. B. Clarke, a medium or large tree, is widely distributed in Himalaya, Madagascar and southern Asia [1]. The fruits have been recorded as Chebulae Fructus in the Chinese Pharmacopoeia, together with those of its original species, *T. chebula*, for the treatment of diarrhea, hemorrhoids, cough, and sore throat [2]. They have also been used traditionally in Tibetan medicines for the treatments of diabetic, tumor, and microbial infection. Our previous phytochemical investigations on the variety reported that the fruit contains rich hydrolyzable tannins, triterpenes, flavonoids, lignans and simple phenolics [3]. As a part of our efforts to search for unique structural constituents from the genus *Terminalia*, two new lignan glucosides termitomenins F and G (**1**–**2**), were isolated from the branches and leaves of *T. chebula* var. *tomentella*, along with five known lignan glucosides (**3**–**7**), six known hydrolyzable tannins (**8**–**13**) and eight simple phenolics (**14**–**21**). Their structures were determined by spectroscopic analyses and comparison of 1D/2D NMR, IR, UV, HRESIMS and calculated ECD analysis. Compounds **1**–**2** are new lignan glucosides with furofuran skeletons. All the isolates were evaluated for their α-glucosidase inhibitory activities. Herein we describe the isolation, structural elucidation, and α-glucosidase inhibitory activities of these compounds.

## Results, Discussion and Conclusion

**Fig. 1:**
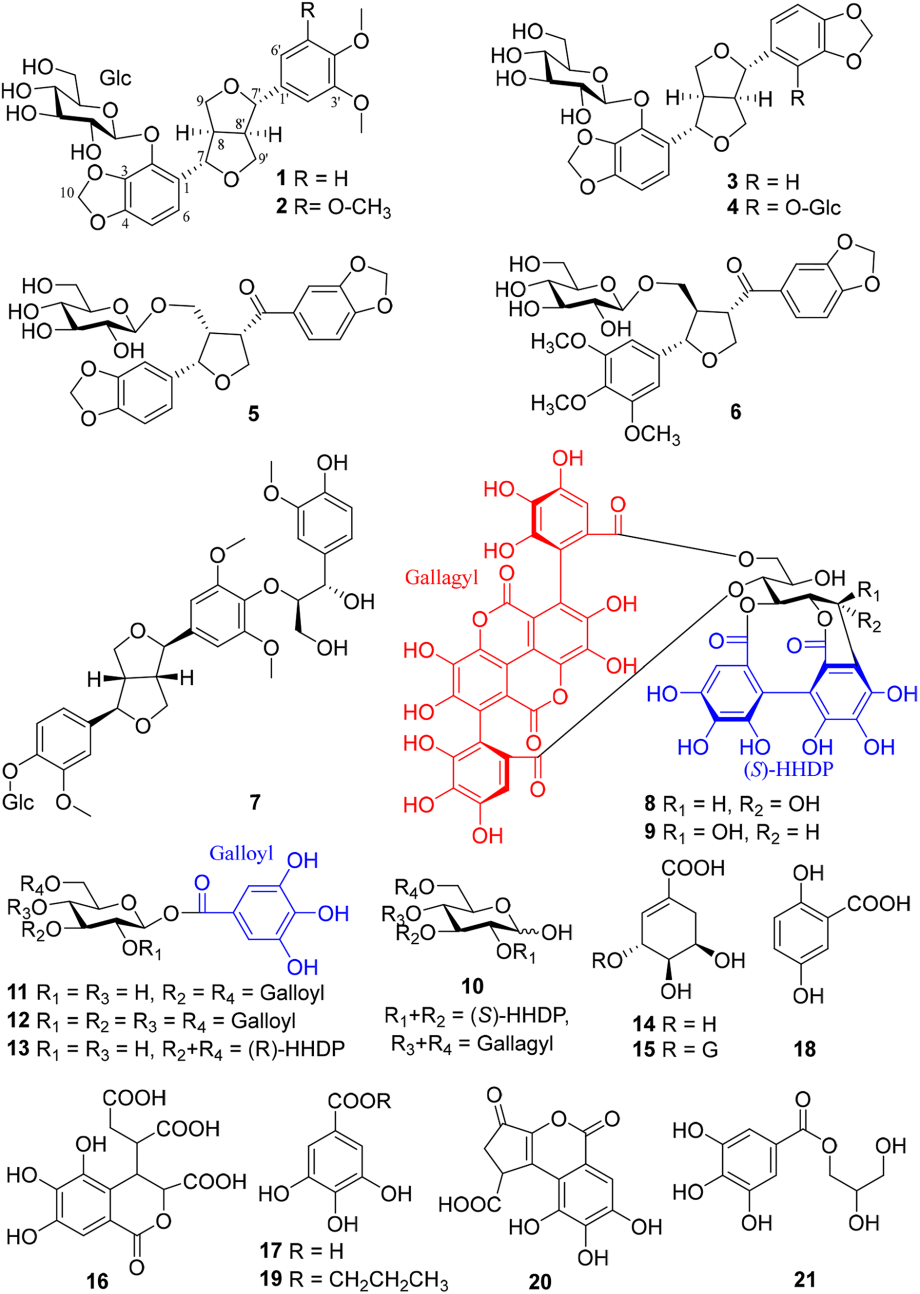
Compounds **1–21** isolated from *Terminalia chebula* var. *tomentella*

The detailed phytochemical investigation on the branches and leaves of *T. chebula* var. *tomentella* afforded two new lignan glucosides (**1**–**2**) (Fig. 1). In addition, 19 known compounds (**3**–**21**) were isolated and identified as five lignan glucosides, termitomenin D (**3**) [3], termitomenin E (**4**) [3], ( +)-(7*S*,8*S*,8′*S*)-9-*O*-[*β*-d-glucopyranoyl] asarininone (**5**) [4], terminaloside Q (**6**) [5], and samsesquinoside (**7**) [6], six hydrolyzable tannins, punicacortein C (**8**) [7], punicacortein D (**9**) [7], punicalagin (**10**) [8], 1,3,6-tri-*O*-galloyl-*β*-d-glucopyranose (**11**) [9], 1,2,3,4,6-penta-*O*-galloyl-*β*-d-glucopyranose (**12**) [9], and corilagin (**13**) [10], and six simple phenolics, shikimic acid (**14**) [11], (−)-5-*O*-galloylshikimic acid (**15**) [12], chebulic acid (**16**) [13], gallic acid (**16**) [14], 2,5-dihydroxybenzoic acid (**18**) [15], propyl gallate (**19**) [16], brevifolincarboxylic acid (**20**) [17], and glycerol-1-gallate (**21**) [18], respectively, on account of comparison of their NMR and HRESIMS data with those recorded in the literatures (Fig. 1). Compounds **8**–**9** were *C*-glycosidic hydrolyzable tannins with one hexahydroxydiphenoyl (HHDP) and one gallagyl group linked to an open-chain glucosyl *C*-1/*O*-2/*O*-3 and *O*-4/*O*-6, rarely found to exist in plants. Compounds **1**–**2**, **6**–**9**, **13**, and **18**–**21** were obtained from the titled plant for the first time, while **3**–**5**, **10**–**12,** and **14**–**17** were also found in the fruits.

**Fig. 2:**
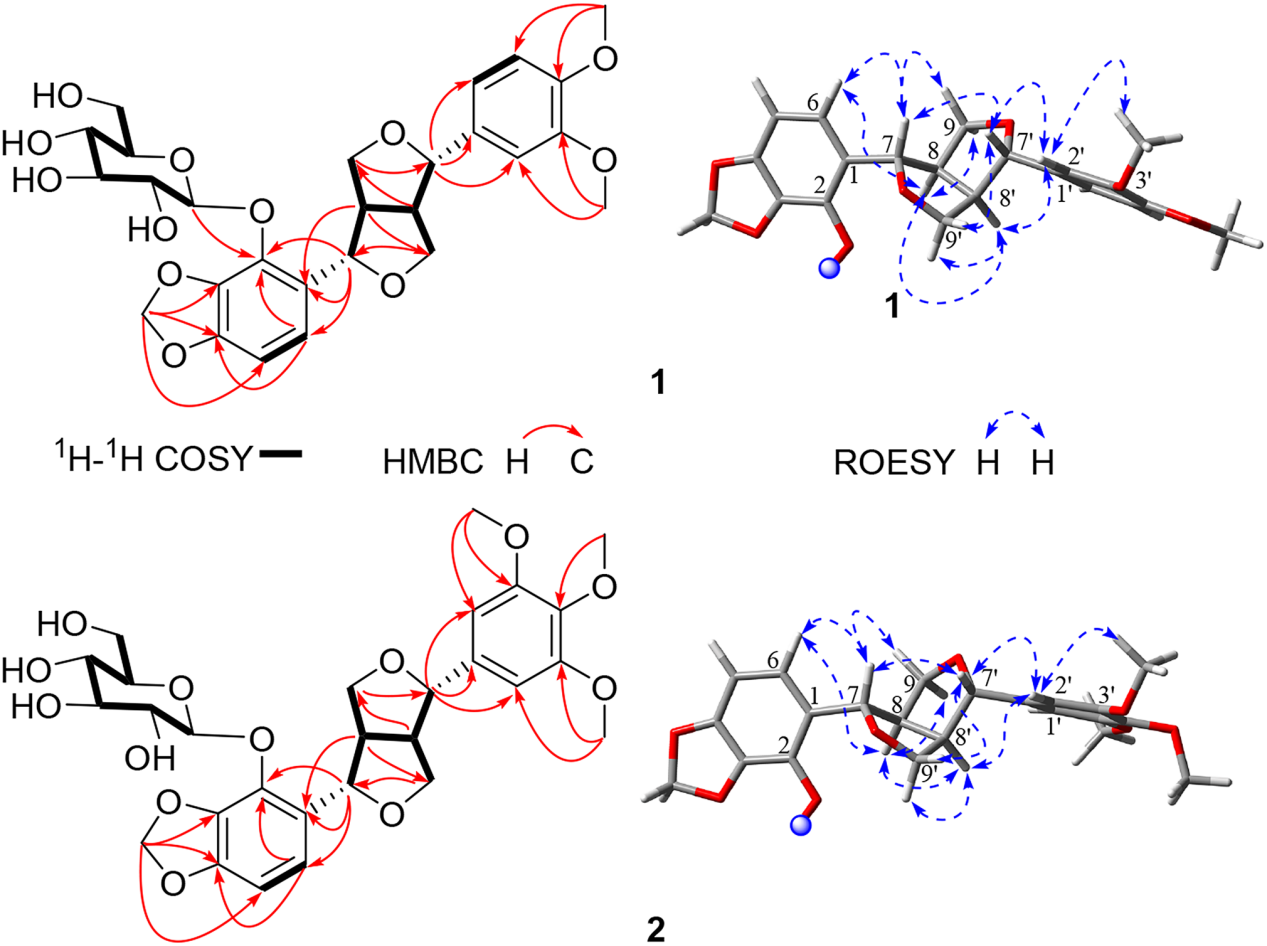
Key ^1^H-^1^H COSY, HMBC and ROESY correlations of compounds **1** and **2**

**Fig. 3:**
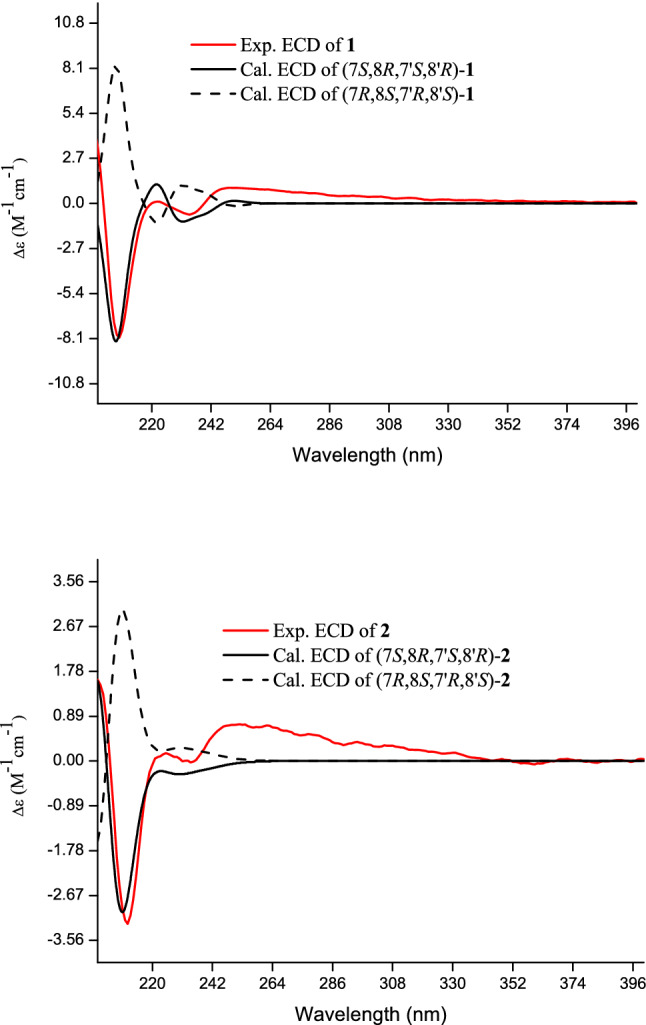
Calculated and experimental ECD spectra of compounds **1** and **2**

Compound **1** was isolated as a yellowish oil. In the HRESIMS^−^, a quasi-molecular ion peak at *m/z* 593.1885 [M + HCOO]^−^ (calcd for 593.1876), indicated a molecular formula of C_27_H_32_O_12_, corresponding to 12 unsaturation degrees. In the ^13^C NMR and DEPT spectra of **1** (Table 1), 27 carbon signals including 12 aromatic carbons (*δ*_C_ 100–160) arising from two benzene rings, one methylenedioxy (*δ*_C_ 102.7), four aliphatic methines (*δ*_C_ 54.8, 55.7) with two oxygen-bearing ones (*δ*_C_ 83.6, 87.4), two oxymethylenes (*δ*_C_ 75.0, 72.4), and two methoxys (2C, *δ*_C_ 56.6) were observed, in addition to a glucosyl moiety (*δ*_C_ 102.5, 78.7, 78.3, 75.6, 71.7, 62.8). The ^1^H NMR spectrum showed the presence of one trisubstituted [*δ*_H_ 6.98 (1H, s, H-2′), 6.93 (2H, brs, H-5′, H-6′)] and one 1,2,3,4-tetrasubstituted [*δ*_H_ 6.54, 6.88 (each 1H, d, *J* = 8.1 Hz, H-5, H-6)] benzene rings. These ^1^H and ^13^C NMR features were closely related to those of termitomenin D (**3**), a furofuran lignan glucoside isolated from the fruits of the titled plant [3], except that one methylenedioxy group in **3** was changed to two methoxy groups (2C, *δ*_C_ 56.6) in **1**. Further HSQC and ^1^H-^1^H COSY experiments could assign completely all the proton signals and their corresponding carbons, together with the furofuran skeleton in **1**. In the HMBC spectrum of **1**, the correlations (Fig. 2) of H-7 (*δ*_H_ 5.19) with C-1 (*δ*_C_ 128.4)/C-2 (*δ*_C_ 138.8)/C-6 (*δ*_C_ 119.7), and H-7′ (*δ*_H_ 4.65) with C-1′ (*δ*_C_ 135.4)/C-2′ (*δ*_C_ 111.3)/*δ*_C_ 120.1 (C-6′) indicated the tetra- and tri- substituted benzene rings were located at C-7 and C-7′, respectively. Moreover, the HMBC correlations of all the *ortho*-coupled aromatic proton at *δ*_H_ 6.88 (d, *J* = 8.1 Hz, H-6), H-7 and the glucosyl anomeric proton (*δ*_H_ 5.39) with C-2 (*δ*_C_ 138.8) demonstrated the glucosyl unit located at C-2 of the tetra-substituted benzene ring, whose C-3 and C-4 linked with a methylenedioxy group, as deduced by the HMBC correlations of the methylenedioxy protons at *δ*_H_ 5.96 and 5.90 (H_2_-10) with C-3 (*δ*_C_ 137.1)/C-4 (*δ*_C_ 150.5), and both H_2_-10 and the *ortho*-coupled aromatic H-6 (*δ*_H_ 6.88) with C-4. Moreover, two additional methoxy protons at *δ*_H_ 3.84 and 3.86 (3′-OCH_3_, 4′-OCH_3_) showed HMBC correlations with *δ*_C_ 150.3 (C-3′) and *δ*_C_ 150.8 (C-4′), respectively. Their locations at C-3′ and C-4′ were further confirmed by the ROESY correlations of 3′-OCH_3_ (*δ*_H_ 3.84) with H-2′ (*δ*_H_ 6.98), and 4′-OCH_3_ (*δ*_H_ 3.86) with H-5′ (*δ*_H_ 6.93). Accordingly, the planar structure of **1** was constructed as shown in Fig. 1. The ROESY correlation of H-8 (*δ*_H_ 3.22) with H-6 (*δ*_H_ 6.88)/H-8′ (*δ*_H_ 3.01), and H-8′ with H-2′ (*δ*_H_ 6.98) indicated that the *cis*-8,8′-fused conformation in **1** and both benzene rings at C-7 and C-7′ were at the same side to H-8 and H-8′, that is, H-7 (*δ*_H_ 5.19) and H-8 (*δ*_H_ 3.22), H-7′ (*δ*_H_ 4.65) and H-8′ (*δ*_H_ 3.01) were at the opposite orientations, respectively. This was also supported by the ROESY correlations of H-7 (*δ*_H_ 5.19) with H-9*β* (*δ*_H_ 4.03), H-8 (*δ*_H_ 3.22) with H-9 α (*δ*_H_ 4.38), H-7′ (*δ*_H_ 4.65) with H-9′*β* (*δ*_H_ 3.92), and H-8′ (*δ*_H_ 3.01) with H-9′ α (*δ*_H_ 4.18). Finally, the ECD calculation of (7*S*,8*R*,7′*S*,8′*R*)-**1** matched well with the experimental ECD curve of **1**, established the absolute configuration of **1** as 7*S*,8*R*,7′*S*,8′*R* (Fig. 3). According to the above mentioned evidence, the structure of compound **1** was determined as shown in Fig. 1 and named as termitomenin F.

**Table 1:** ^1^H (600 MHz) and ^13^C (150 MHz) NMR data of **1** in CD_3_OD (*δ* in ppm, *J* in Hz)

No.	*δ*_C_	*δ*_H_, mult. (*J*)	No.	*δ*_C_	*δ*_H_, mult. (*J*)
1	128.4		5′	112.9	6.93 brs
2	138.8		6′	120.1	6.93 brs
3	137.1		7′	87.4	4.65 d (6.2)
4	150.5		8′	55.7	3.01 m
5	103.7	6.54 d (8.1)	9′	72.4	4.18 dd (9.1, 6.2)
6	119.7	6.88 d (8.1)			3.92 dd (9.1, 3.5)
7	83.6	5.19 d (4.3)	3′-OCH_3_	56.6	3.84 s
8	54.8	3.22 m	4′-OCH_3_	56.6	3.86 s
9	75.0	4.38 dd (8.9, 7.7)	Glc-1″	102.5	5.39 d (7.6)
		4.03 dd (8.9, 5.1)	2″	75.6	3.43 m
10	102.7	5.96 d (1.2), 5.90 d (1.2)	3″	78.3	3.35 m
1′	135.4		4″	71.7	3.34 m
2′	111.3	6.98 s	5″	78.7	3.35 m
3′	150.3		6″	62.8	3.85 m,
4′	150.8				3.65 dd (12.1, 5.2)

Compound **2** was isolated as a yellow oil. In the HRESIMS^−^, a quasi-molecular ion peak appeared at *m/z* 613.1700 [M + Cl]^−^ (calcd for 613.1693 [M + Cl]^−^), indicating a molecular formula of C_28_H_34_O_13_, corresponding to 12 unsaturation degrees. In the ^13^C NMR and DEPT spectra of **2** (Table 2) displayed the presence of 28 carbon signals including 12 aromatic carbons (*δ*_C_ 100–160 ppm) arising from two benzene rings, one methylenedioxy (*δ*_C_ 102.7), four aliphatic methines (*δ*_C_ 54.8, 56.1) with two oxygen-bearing ones (*δ*_C_ 83.6, 87.5), two oxymethylenes (*δ*_C_ 75.2, 72.4), and three methoxys [*δ*_C_ 56.8 (× 2), 61.2], and six carbon signals (*δ*_C_ 102.5, 78.7, 78.3, 75.6, 71.7, 62.7) from a glucosyl moiety. The ^1^H NMR spectrum of **2** showed characteristic signals of two *ortho*-coupled aromatic protons at *δ*_H_ 6.53, 6.88 (each 1H, d, *J* = 8.1 Hz, H-5, H-6), one two-proton singlet at *δ*_H_ 6.68 (2H, s, H-2′, H-6′), one anomeric proton at *δ*_H_ 5.39 (1H, *J* = 7.5 Hz, H-1"), and a set of signals from *δ*_H_ 2.99 to 5.18 due to a furofuran skeleton, a glucosyl and a methylenedioxy moieties. The ^13^C and ^1^H NMR features of **2** were quite similar to those of **1**, except that **2** had one more methoxy group (*δ*_C_ 61.2) than **1**. Compound **2** was also a furofuran lignan glucoside. Instead of the 1,3,4-trisubsituted benzene ring in **1**, a symmetric 1,3,4,5-tetrasubstituted benzene ring appeared in **2**. These were further confirmed by the 2D NMR experiment. In the HMBC spectrum of **2**, *δ*_H_ 3.84 (6H, s, 3′-OCH_3_, 5′-OCH_3_) and 3.74 (3H, s, 4′-OCH_3_) showed respectively correlations with *δ*_C_ 154.8 (C-3′, C-5′) and 138.6 (C-4′), indicating the three methoxy groups were at C-3′, C-4′ and C-5′ (Fig. 2). Other HMBC correlations also supported the planar structure of **2** as shown in Fig. 1. Moreover, the ROESY experiment showed that the relative configuration of **2** was the same as that of **1** (Fig. 2). In which, the ROESY correlations of H-8 (*δ*_H_ 3.20) with H-6 (*δ*_H_ 6.88) / H-8′ (*δ*_H_ 2.99), and H-8′ with H-2′ (*δ*_H_ 6.68) / H-6′ indicated that H-7 and H-8, and H-7′ and H-8′ were at the opposite orientations, respectively. This was confirmed by the ROESY correlations of H-7 with H-9*β*, H-8 with H-9 α, H-7′ with H-9′*β*, and H-8′ with H-9′α. Eventually, the ECD calculation of (7*S*,8*R*,7′*S*,8′*R*)-**2** matched well with the experimental ECD curve of **2**, revealed the absolute configuration of **2** as 7*S*,8*R*,7′*S*,8′*R* (Fig. 3). According to the aforementioned evidence, the structure of compound **2** was determined as shown in Fig. 1 and named as termitomenin G.

**Table 2:** ^1^H (600 MHz) and ^13^C (150 MHz) NMR data of **2** in CD_3_OD (*δ* in ppm, *J* in Hz)

No.	*δ*_C_	*δ*_H_, mult. (*J*)	No.	*δ*_C_	*δ*_H_, mult. (*J*)
1	128.4		4′	138.6	
2	138.8		7′	87.5	4.66 d (6.1)
3	137.1		8′	56.1	2.99 dtd (9.7, 6.1, 3.5)
4	150.5		9′	72.4	4.19 dd (9.2, 6.1)
5	103.7	6.53 d (8.1)			3.96 dd (9.2, 3.5)
6	119.7	6.88 d (8.1)	3′,5′-OCH_3_	56.8	3.84 s
7	83.6	5.18 d (4.3)	4′-OCH_3_	61.2	3.74 s
8	54.8	3.20 m	Glc-1″	102.5	5.39 d (7.5)
9	75.2	4.39 dd (9.0, 7.4)	2″	75.6	3.41 m
		4.05 dd (9.0, 5.1)	3″	78.3	3.34 m
10	102.7	5.95 d (1.0), 5.89 d (1.0)	4″	71.7	3.45 m
1′	139.0		5″	78.7	3.35 m
2′, 6′	104.4	6.68 s	6″	62.7	3.82 m
3′, 5′	154.8				3.64 dd (11.9, 5.1)

Compounds **1**–**21** were evaluated for their hypoglycemic activity with quercetin as positive controls [19]. As shown in Table 3, only the hydrolyzable tannins **8**–**13** showed α-glucosidase inhibitory activities with the IC_50_ values from 0.10 to 3.12 μM, stronger than the positive control, quercetin (IC_50_ = 9.38 ± 0.33 μM), while the other compounds did not show obvious inhibitory activity at a concentration of 50 μM. Among them, compound **12** with five galloyl group in molecular showed the strongest effect on α-glucosidase, followed with **10** > **9** > **8** > **11** > **13** by the order of activity strength. When the open-chain glucosyl moiety was fixed by a *C*-glycosidic bond with a HHDP group (**8** and **9**), it displayed less activity than that one (**10**) with a free glucosyl anomeric center. Moreover, compound **11** with nine phenol OHs arising from three galloyl groups showed a little bit stronger activity than **13** with the same nine phenol OHs from one galloyl and one HHDP groups. The results suggested that galloyl and HHDP groups may play a vital role for the α-glucosidase inhibitory activity of these compounds (**8**–**13**), much stronger than those lignan glucosides and phenolic compounds.

**Table 3:** α-Glucosidase inhibitory activity of compounds **8**–**13** from the branches and leaves of *Terminalia chebula* var. *tomentella*^*a*^

Sample	IC_50_ (μM)^*b*^
Quercetin^*c*^	9.38 ± 0.33
**8**	1.96 ± 0.01
**9**	1.72 ± 0.07
**10**	0.27 ± 0.01
**11**	2.28 ± 0.07
**12**	0.10 ± 0.01
**13**	3.12 ± 0.23

^a^Values represent means ± SD (n = 3). ^b^IC_50_ = one-half maximal inhibitory concentration to α-glucosidase. ^c^Positive control

## Experimental Section

### General Experimental Procedures

One- and two-dimensional NMR spectra were determined on methanol-*d*_4_ or D_2_O with Bruker Ascend 600 and AV-800 spectrometers. Coupling constants were expressed in hertz (Hz), and chemical shifts were recorded in a *δ* (parts per million, ppm) scale with TMS (Bruker, Zurich, Switzerland) as an internal standard. ESI mass spectra were measured on a VG Auto Spec300 spectrometer. High-resolution electrospray ionization mass (HRESIMS) spectra were measured on an API QSTAR Pular-1 spectrometer. Optical rotations were taken on JASCO P-1020 digital polarimeter. IR spectra were recorded on Bio-Rad FTS 135 series spectrometer with KBr pellets. UV spectra were given on and UV-2410PC Shimadzu spectrometer. Column chromatography (CC) was performed on 25–100 μm Sephadex LH-20 (Pharmacia Fine Chemical Co., Ltd., Uppsala, Sweden), 75–100 μm MCI-gel CHP20P (Mitsubishi Chemical Co. Ltd., Tokyo, Japan), and 100–150 mesh silica gel (Qingdao Marine Chemical, Inc., Qingdao, China). Thin-layer chromatography (TLC) was performed on precoated 0.20–0.25 mm thick silica gel GF254 plates (Qingdao Haiyang Chemical Co., Qingdao, China), Agilent series 1260 (Agilent Technologies) were used for semi-preparative HPLC with an Agilent ZORBAX SB-C18 column (5 μm, 250 mm × 9.4 mm). Acetonitrile (chromatographic grade) were purchased from Merck (Darmstadt, FR, Germany). Water was purified in a Milli-Q apparatus (Millipore). 4-Nitrophenyl-α-D-glucopyranoside (PNPG) was procured from America Sigma Chemical Co. α-Glucosidase and quercetin were purchased from Sigma Chemical (Merck KGaA, Darmstadt, Germany). Potassium phosphate buffer solution (PPBS) was purchased from Shanghai Xilong Biochemical Technology Co. Ltd. 96-well plates was obtained from Qingdao Haiyang Chemical Co., Ltd.

### Plant Material

The branches and leaves of *Terminalia chebula* var. *tomentella* (Kurz) C. B. Clarke (Combretaceae), identified by Dr. En-De Liu from Kunming Institute of Botany, Chinese Academy of Sciences, were collected in Lincang County, Yunnan Province, P. R. China, on November 2017. A standard sample (KIB-1-Z-20171102) has been deposited in the State Key Laboratory of Phytochemistry and Plant Resource in West China of Kunming Institute of Botany.

### Extraction and Isolation

The air-dried branches and leaves of *T. chebula* var. *tomentella* (10 kg) were crushed and extracted by 60% aqueous acetone solution at room temperature. After removal of organic solvent by rotary evaporator at 45 °C under reduced pressure, the crude aqueous extract (3 L) was applied to a Diaion HP-20 column chromatography (water–methanol 1:0–0:1) to obtain three fractions, Fr. I—III. Fr. I (355.9 g) was applied to repeated CC over Sephadex LH-20, MCI-gel CHP20P, Toyopearl HW-40F, and MCI-gel CHP20P, eluting with water–methanol (1:0–0:1), to furnish compounds **7** (5 mg), **8** (3 mg), **9** (3 mg), **10** (55 mg), and **11** (15 mg). Fr. II (163.9 g) was applied to repeated CC over Sephadex LH-20, MCI-gel CHP20P, Toyopearl HW-40F, and Rp18, eluting with water–methanol (1:0–0:1), to obtain compounds **3** (5 mg), **4** (2 mg), **5** (2 mg), and **14** (3 mg). Fr. III (993.4 g) was separately chromatographed over Sephadex LH-20, MCI-gel CHP20P, Toyopearl HW-40F water–methanol (1:0 − 3:7), and silica gel CC eluting with CHCl_3_/MeOH 15:1–1:1, to yield compounds **1** (5 mg), **2** (5 mg), **6** (20 mg), **12** (13 mg), **13** (10 mg), **15** (16 mg), **16** (4 mg), **17** (3 mg), **18** (4 mg), **19** (5 mg), **20** (0.9 mg), and **21** (6 mg).

#### Termitomenin F (1)

Colorless oil, [*α*]^22^_D_ ‒ 13.2 (*c* 0.09, MeOH). UV (MeOH) *λ*_max_ (log ε) 205 (3.65) nm. IR (KBr) *ν*_max_ 3399, 2923, 2875, 1467, 1260 cm^−1^. ^1^H (600 MHz) and ^13^C (150 MHz) NMR (in methanol-*d*_4_) data, see Table 1. HRESIMS (negative-ion mode) *m/z* 593.1885 [M + 45 (COOH)]^−^ (calcd for C_28_H_33_O_14_, 593.1876).

#### Termitomenin G (2):

Colorless oil, [*α*]^22^_D_ ‒ 4.1 (*c* 0.075, MeOH). UV (MeOH) *λ*_max_ (log ε) 205 (3.71) nm. IR (KBr) *ν*_max_ 3409, 2925, 1630, 1465 cm^−1^. ^1^H (600 MHz) and ^13^C (150 MHz) NMR (in methanol-*d*_4_) data, see Table 2. HRESIMS (negative-ion mode) *m/z* 613.1700 [M + Cl]^−^ (calcd for C_28_H_34_O_13_Cl, 613.1693).

### ECD Computational Details

Conformational analyzes were conducted by random searching in the Sybyl Software X 2.0 using the MMFF-94S force field with an energy shortoff of 2.0 kcal mol^−1^. The results indicated six lowest energy conformers for compounds **1** and **2**. Then, the conformers were re-optimized using DFT at the PBE0-D3(BJ)/def2-SVP level in Methanol using the polarizable conductor calculation model by the ORCA-4.2.1 program [20]. The energies, rotational strengths (velocity), and oscillator strengths of the first 60 electronic excitations were calculated using the TDDFT methodology at the PBE0/def-2-TZVP level in Methanol. The electronic circular dichroism spectra were simulated by the overlapping Gaussian function (half the bandwidth at 1/e peak height, *σ* = 0.30 for all) [21]. Eventually, the electronic circular dichroism spectra of compounds **1** and **2** were gained by weighing the Boltzmann distribution ratio of each geometric conformation.

### α-Glucosidase Inhibitory Activity

The α-glucosidase inhibitor screening assay was conducted as reported previously and slightly modified [22]. Quercetin was used as positive control. 4-Nitrophenol-α-d-glucopyranoside (PNPG) was used as an enzyme inhibitor screening model. α*-*glucosidase solution (0.025 U·mL^−1^), PNPG (0.1 M), phosphate buffer (pH 6.8) and test samples (50 μM) were incubated in 96-well plates at 37 °C for 1 h. A microplate reader was recorded by the absorbance at 405 nm. No enzyme as the blank readings were subtracted from each well and compared to the control. In this assay, all reactions were performed in triplicate. The α-glucosidase inhibitory activity was presented as inhibition ratio. The formula to calculate the inhibition rate is as follows: inhibition rate (%) = (1 ‒ OD _experimental_ 405 nm/OD _blank_ 405 nm) × 100%, and IC_50_ values were calculated according to the Reed and Muench method [23]*.*

## Conclusion

In conclusion, 21 phenolic compounds (**1**–**21**) including seven lignan glucosides (**1**–**7**), six hydrolyzable tannins (**8**–**13**) and eight simple phenolics (**14**–**21**) were isolated and determined from the branches and leaves of *T. chebula* var. *tomentella*. Compounds **1** and **2** were two new lignan glucosides with a furofuran skeleton, while **8** and** 9** were *C*-glycosidic hydrolyzable tannins with one HHDP and one gallagyl groups linked to an open-chain glucosyl *C*-1/*O*-2/*O*-3 and *O*-4/*O*-6, respectively, rarely found to exist in plants. Compounds **1**–**2**, **6**–**9**, **13**, and **18**–**21** were obtained from the titled plant for the first time, while **3**–**5**, **10**–**12**, and **14**–**17** were also find in the fruits. The hydrolyzable tannins **8**–**13** exhibited strong α-glucosidase inhibitory activities.

## Supplementary Information

Below is the link to the electronic supplementary material.

Supplementary file1 (DOCX 4492 kb)
