# Supplementary material for: Termitomenins F and G, Two New Lignan Glucosides from Terminalia chebula var. tomentella (Kurz) C. B. Clarke
Source: Nat Prod Bioprospect. 2021 Jun 10;11(5):565–72. doi: 10.1007/s13659-021-00314-z (PMC8390638; doi:10.1007/s13659-021-00314-z)
Supplement: Supplementary file 1 — Supplementary file1 (DOCX 4492 kb) [file 13659_2021_314_MOESM1_ESM.docx]

Supporting Information

Termitomenins F and G, two new lignans from *Terminalia chebula* var. *tomentella* (Kurz) C. B. Clarke

Jun Yin^1,2^ **·** Hong-Tao Zhu^1^ **·** Man Zhang^1^ **·** Dong Wang^1^ **·** Chong-Ren Yang^1^ **·** Ying-Jun Zhang^1,3^

Affiliations

^1^ State Key Laboratory of Phytochemistry and Plant Resources in West China, Kunming Institute of Botany, Chinese Academy of Sciences, Kunming 650204, People’s Republic of China

^2^ University of Chinese Academy of Sciences, Beijing 100049, People’s Republic of China

^3^ Yunnan Key Laboratory of Natural Medicinal Chemistry, Kunming Institute of Botany, Chinese Academy of Sciences, Kunming 650201, People’s Republic of China

Correspondence

Prof. Dr. Ying-Jun Zhang

State Key Laboratory of Phytochemistry and Plant Resources in West China

Kunming Institute of Botany, Chinese Academy of Sciences

Kunming 650201

P. R. China

Tel/ Fax: +86-871-65223235

E-mail: [zhangyj@mail.kib.ac.cn](mailto:zhangyj@mail.kib.ac.cn)

**Table of contents**

**Contents Page**

[**Fig. S1** ^1^H NMR spectrum of compound **1** in CD_3_OD 3](#_Toc70431658)

[**Fig. S2** ^13^C and DEPT NMR spectrum of compound **1** in CD_3_OD 4](#_Toc70431659)

[**Fig. S3** ^1^H-^1^H COSY spectrum of compound **1** in CD_3_OD 5](#_Toc70431660)

[**Fig. S4** HMBC spectrum of compound **1** in CD_3_OD 6](#_Toc70431661)

[**Fig. S5** HSQC spectrum of compound **1** in CD_3_OD 7](#_Toc70431662)

[**Fig. S6** ROESY spectrum of compound **1** in CD_3_OD 8](#_Toc70431663)

[**Fig. S7** The (-)-ESIMS spectroscopic data of compound **1** 9](#_Toc70431664)

[**Fig. S8** The (-)-HRESIMS spectroscopic data of compound **1** 10](#_Toc70431665)

[**Fig. S9** The IR spectrum of compound **1** 11](#_Toc70431666)

[**Fig. S10** The UV spectrum of compound **1** in CD_3_OD 12](#_Toc70431667)

[**Fig. S11** The ECD spectrum of compound **1** in CD_3_OD 13](#_Toc70431668)

[**Fig. S12** The ^1^H NMR spectrum of compound **2** in CD_3_OD 14](#_Toc70431669)

[**Fig. S13** The ^13^C and DEPT NMR spectrum of compound **2** in CD_3_OD 15](#_Toc70431670)

[**Fig. S14** ^1^H-^1^H COSY spectrum of compound **2** in CD_3_OD 16](#_Toc70431671)

[**Fig. S15** HMBC spectrum of compound **2** in CD_3_OD 17](#_Toc70431672)

[**Fig. S16** HSQC spectrum of compound **2** in CD_3_OD 18](#_Toc70431673)

[**Fig. S17** ROESY spectrum of compound **2** in CD_3_OD 19](#_Toc70431674)

[**Fig. S18** The (-)-ESIMS spectroscopic data of compound **2** 20](#_Toc70431675)

[**Fig. S19** The (-)-HRESIMS spectroscopic data of compound **2** 21](#_Toc70431676)

[**Fig. S20** The IR spectrum of compound **2** 22](#_Toc70431677)

[**Fig. S21** The UV spectrum of compound **2** in CD_3_OD 23](#_Toc70431678)

[**Fig. S22** The ECD spectrum of compound **2** in CD_3_OD 24](#_Toc70431679)


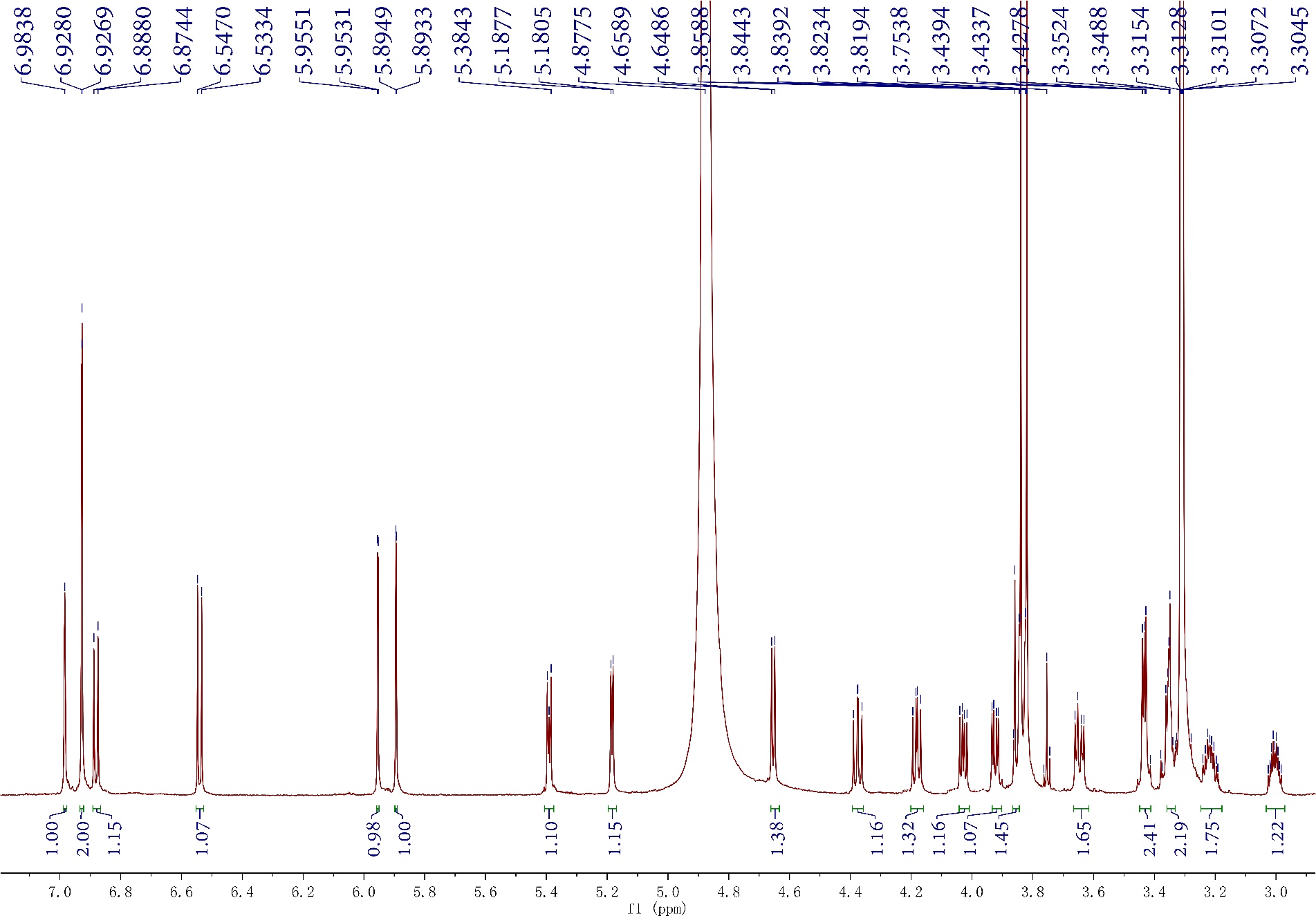


## **[Fig. S1](#_Toc61201451)** ^[1](#_Toc61201451)^[H NMR spectrum of compound](#_Toc61201451) **[1](#_Toc61201451)** [in CD](#_Toc61201451)_[3](#_Toc61201451)_[OD](#_Toc61201451)


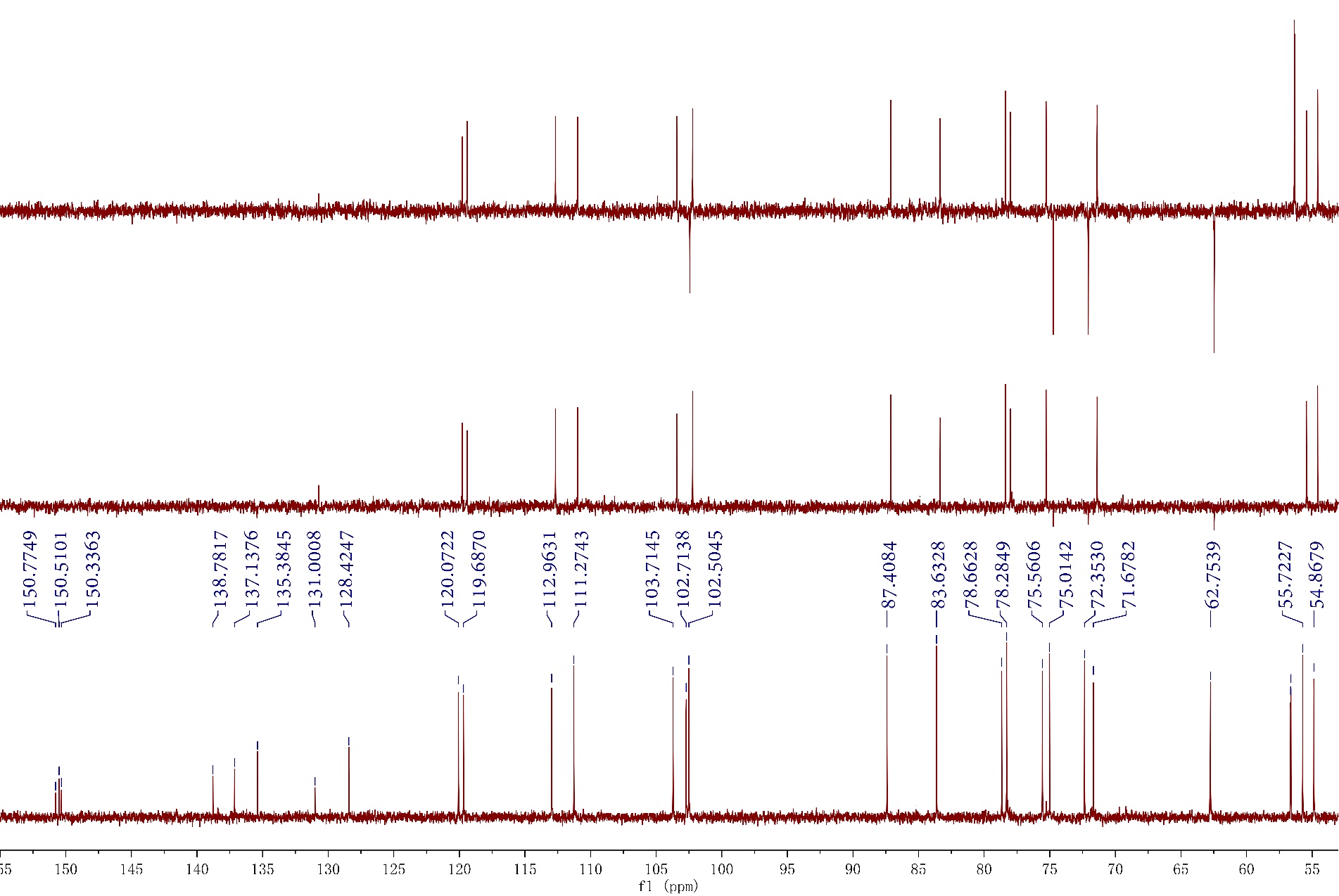


## [**Fig. S2** ^13^C and DEPT NMR spectrum of compound **1** in CD_3_OD](#_Toc61201452)


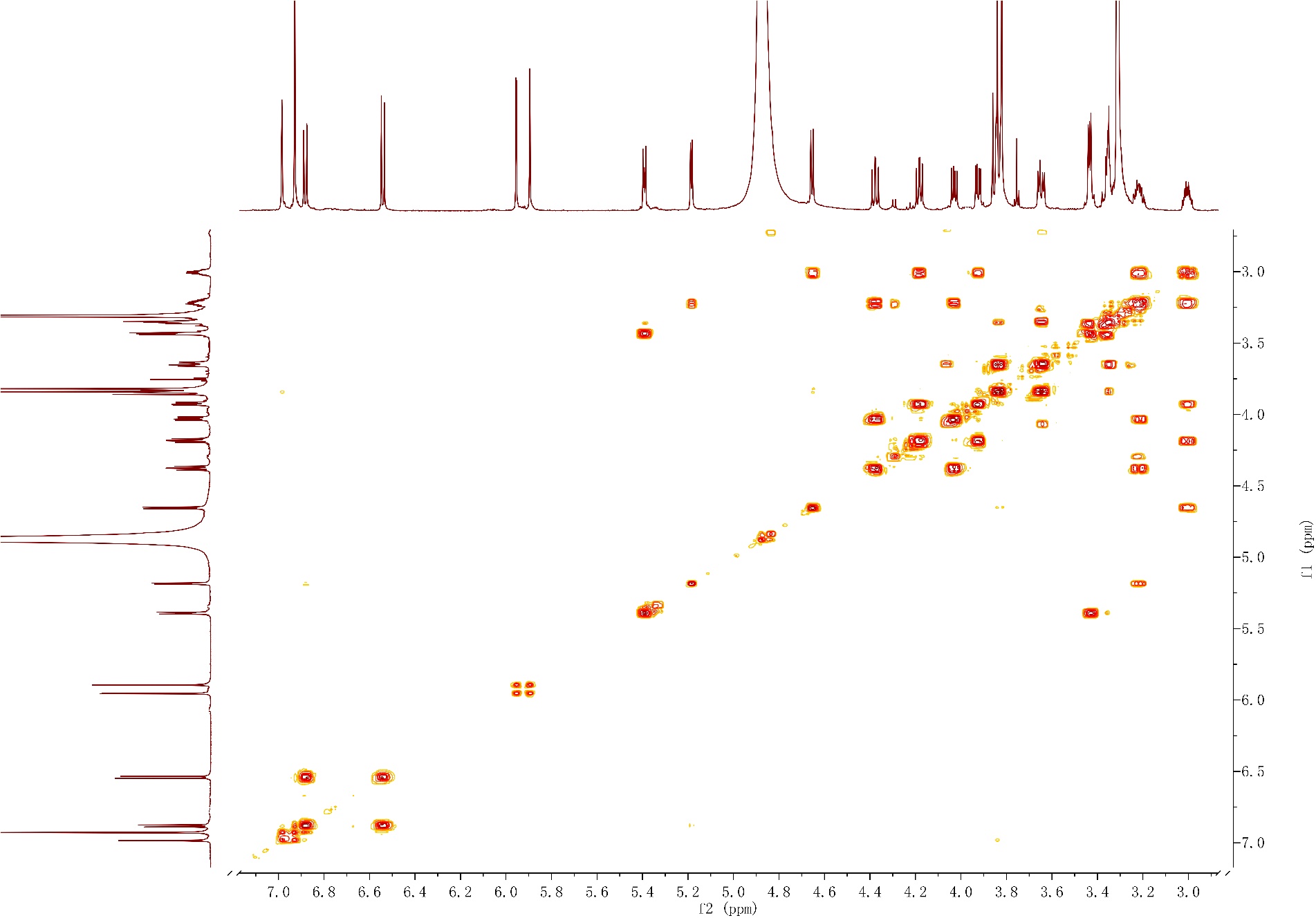


## [**Fig. S3** ^1^H-^1^H COSY spectrum of compound **1** in CD_3_OD](#_Toc61201455)


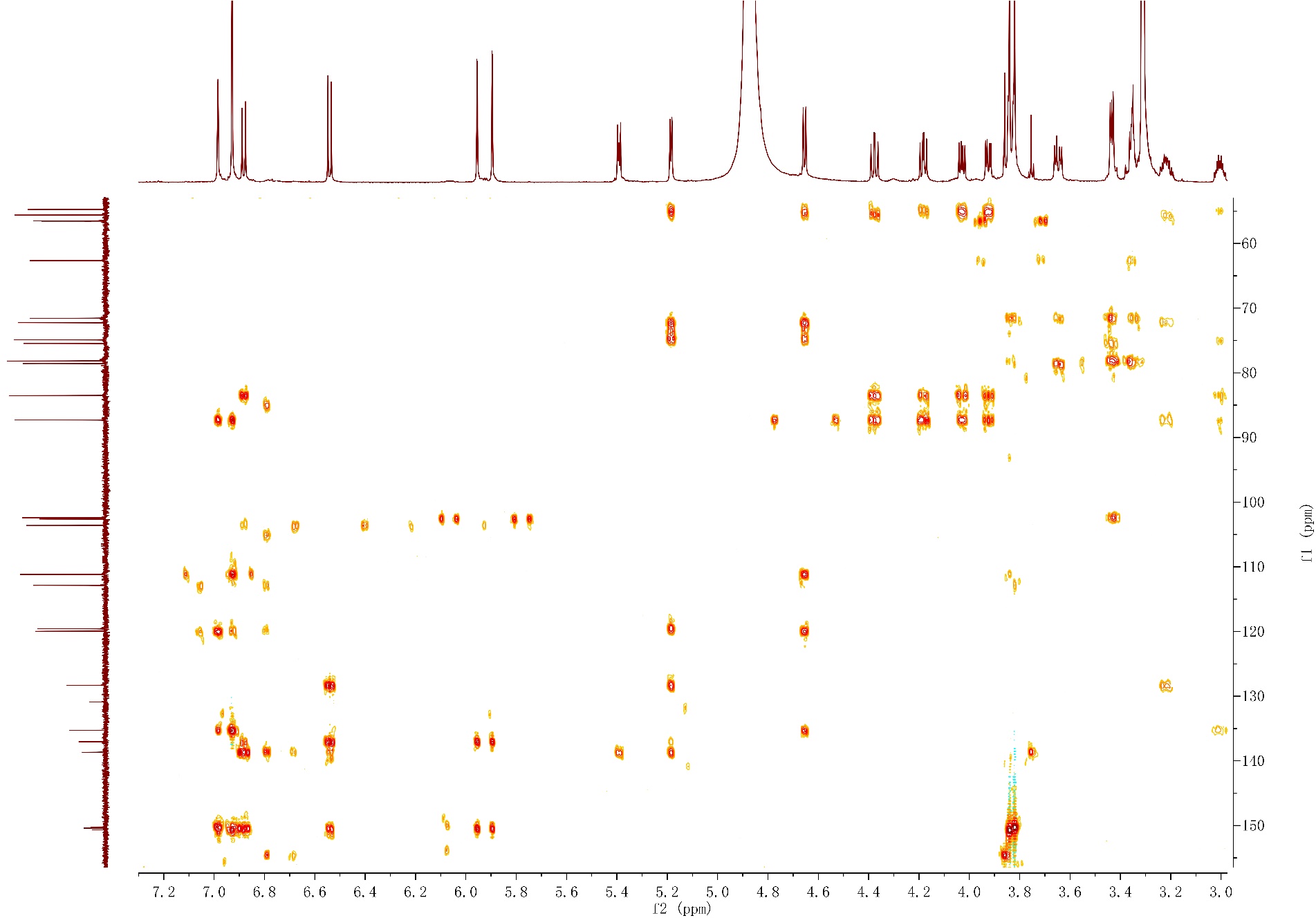


## [**Fig. S4** HMBC spectrum of compound **1** in CD_3_OD](#_Toc61201456)


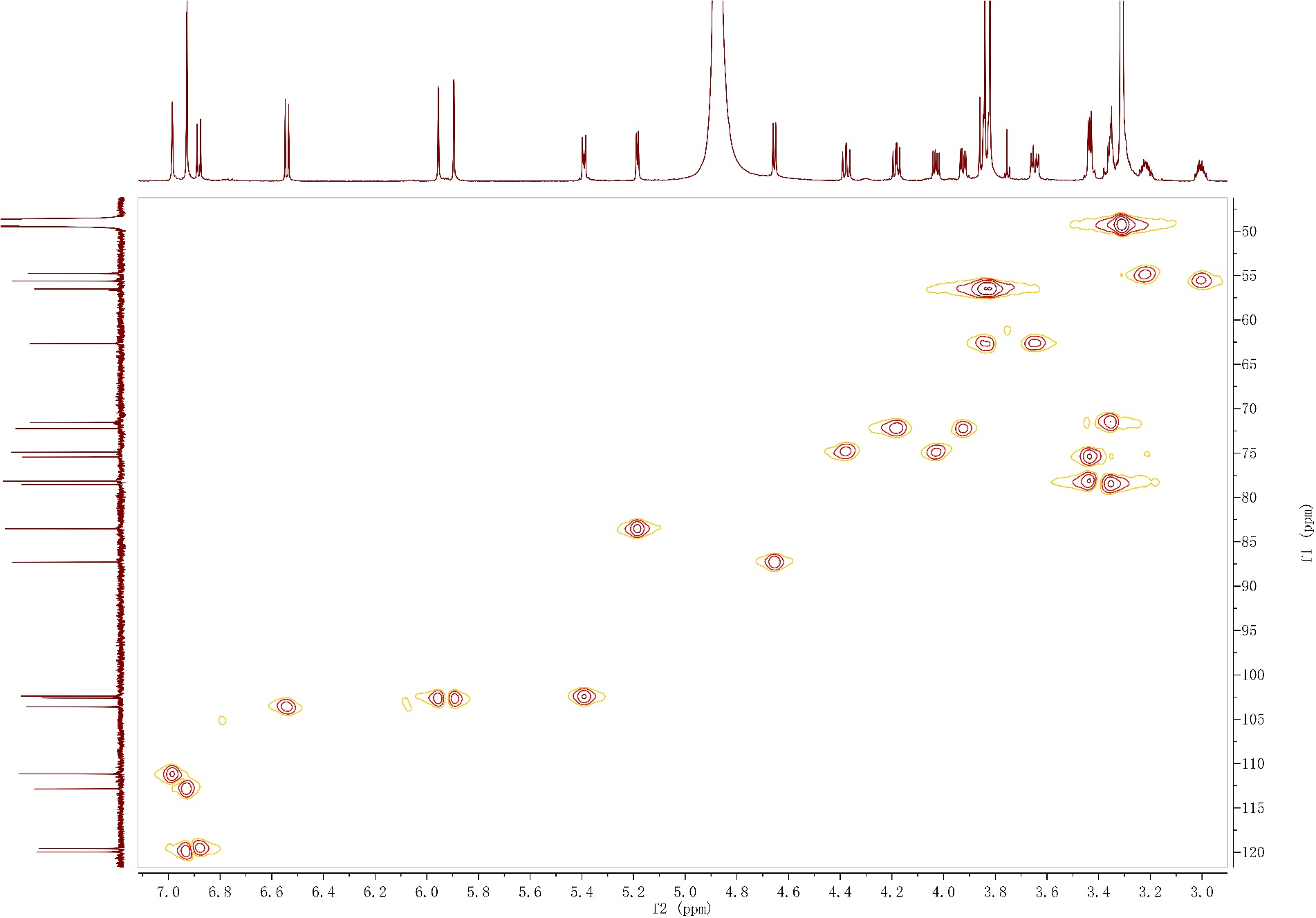


## [**Fig. S5** HSQC spectrum of compound **1** in CD_3_OD](#_Toc61201454)


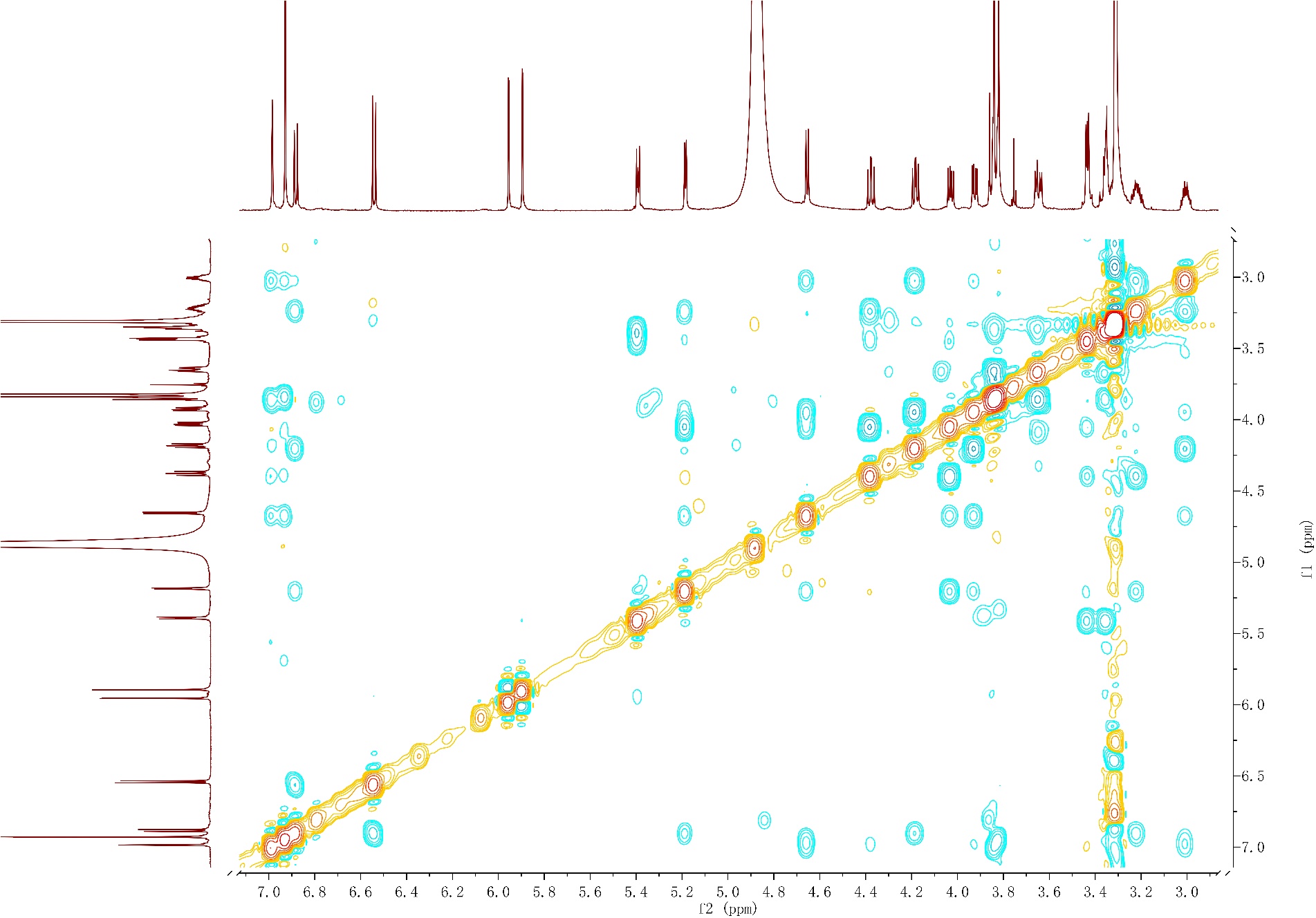


## [**Fig. S6** ROESY spectrum of compound **1** in CD_3_OD](#_Toc61201454)


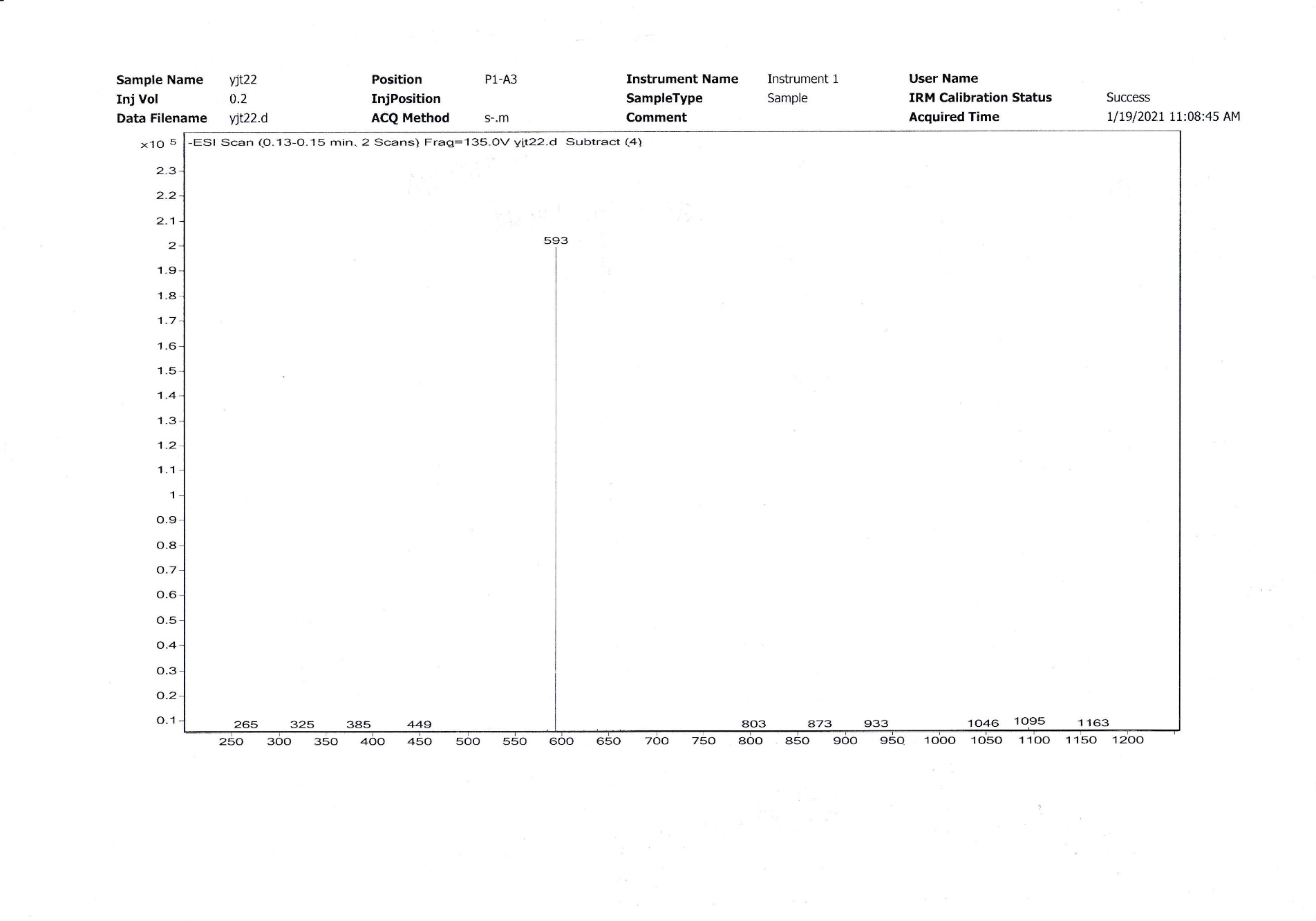


## [[**Fig. S7** The (-)-ESIMS spectroscopic data of compound **1**](#_Toc61201450)](#_Toc61201456)


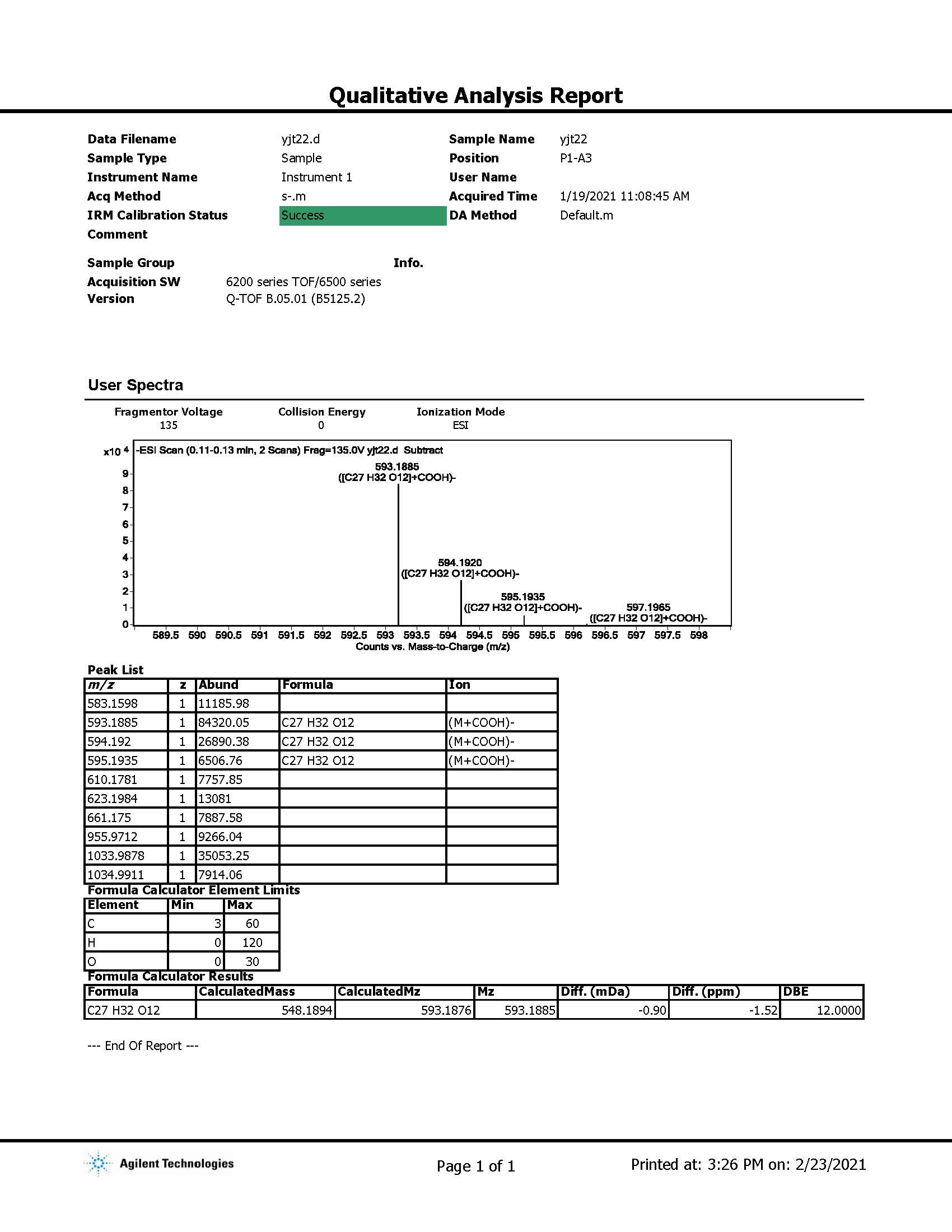


## [Fig. S8 The (-)-HRESIMS spectroscopic data of compound 1](#_Toc61201450)

## **Fig. S9** The IR spectrum of compound **1**


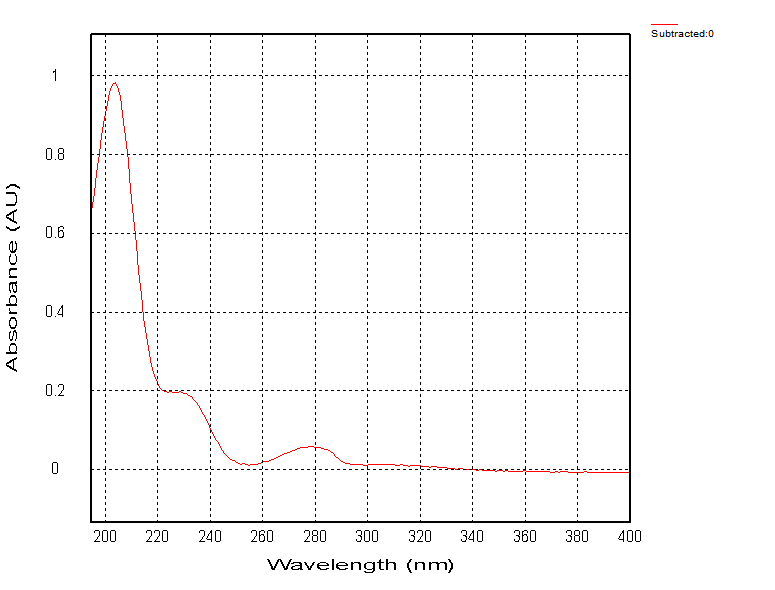


## **Fig. S10** The UV spectrum of compound **1** in CD_3_OD

## **Fig. S11** The ECD spectrum of compound **1** in CD_3_OD


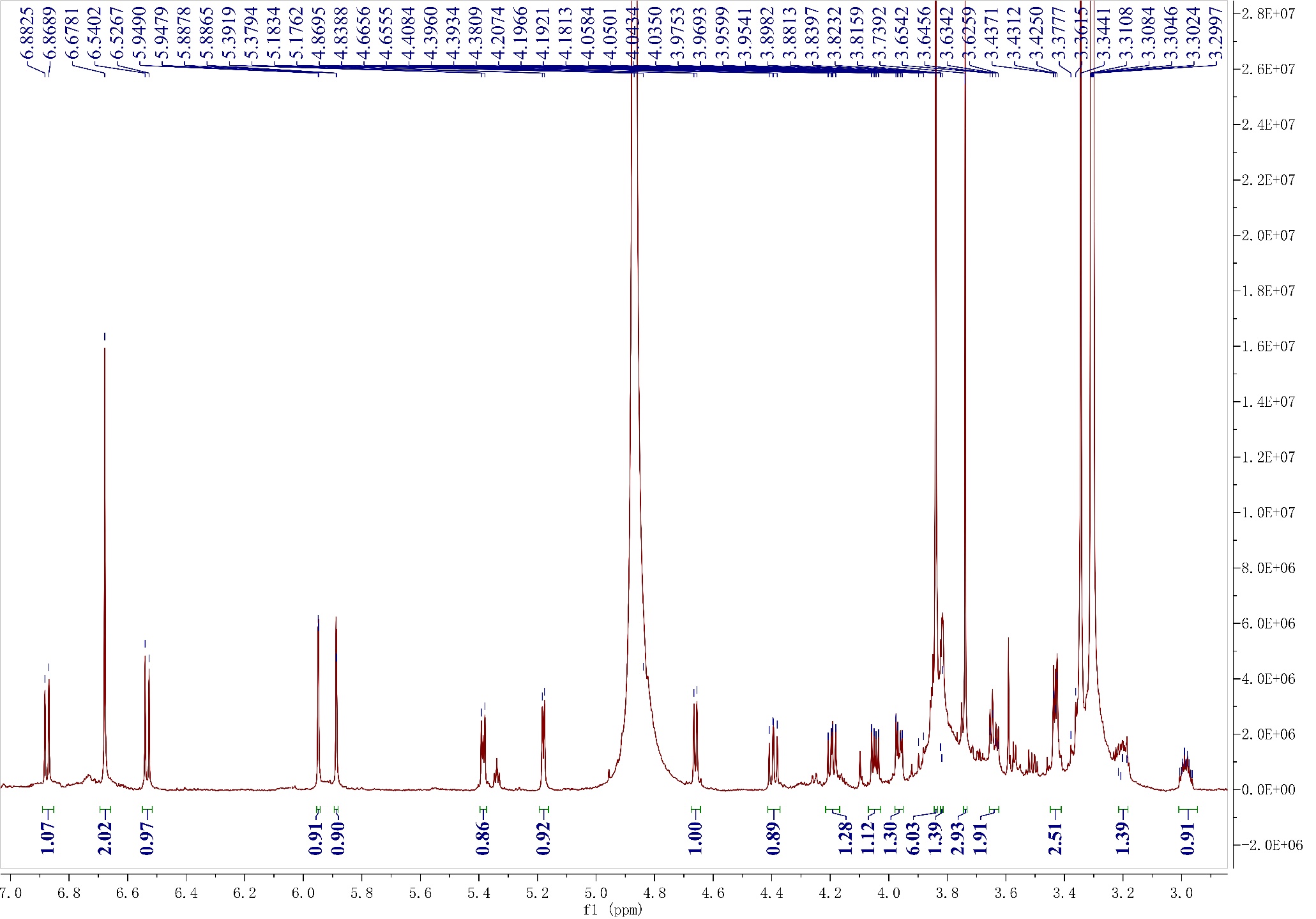


## **Fig. S12** The ^1^H NMR spectrum of compound **2** in CD_3_OD


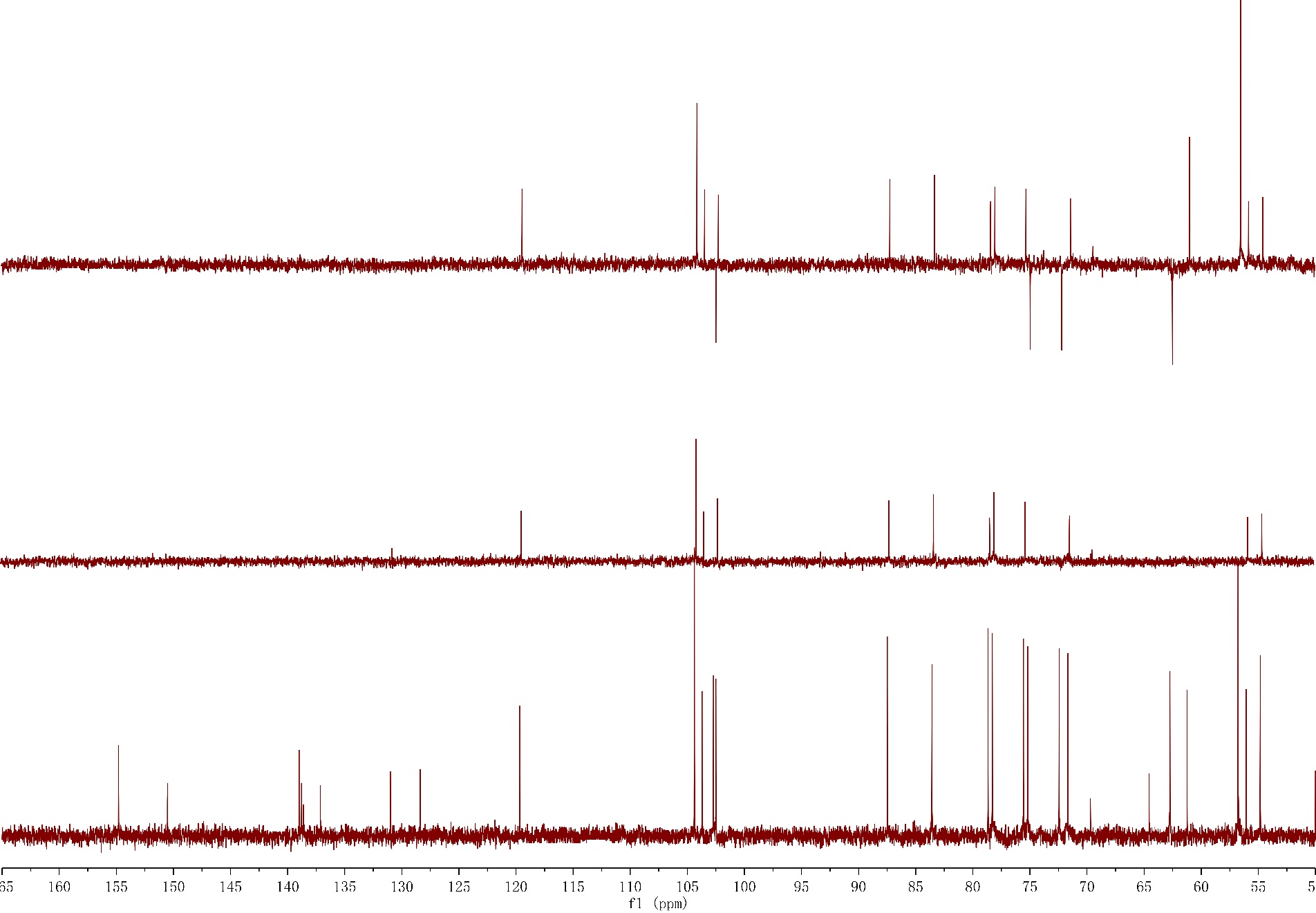


## **Fig. S13** The ^13^C and DEPT NMR spectrum of compound **2** in CD_3_OD


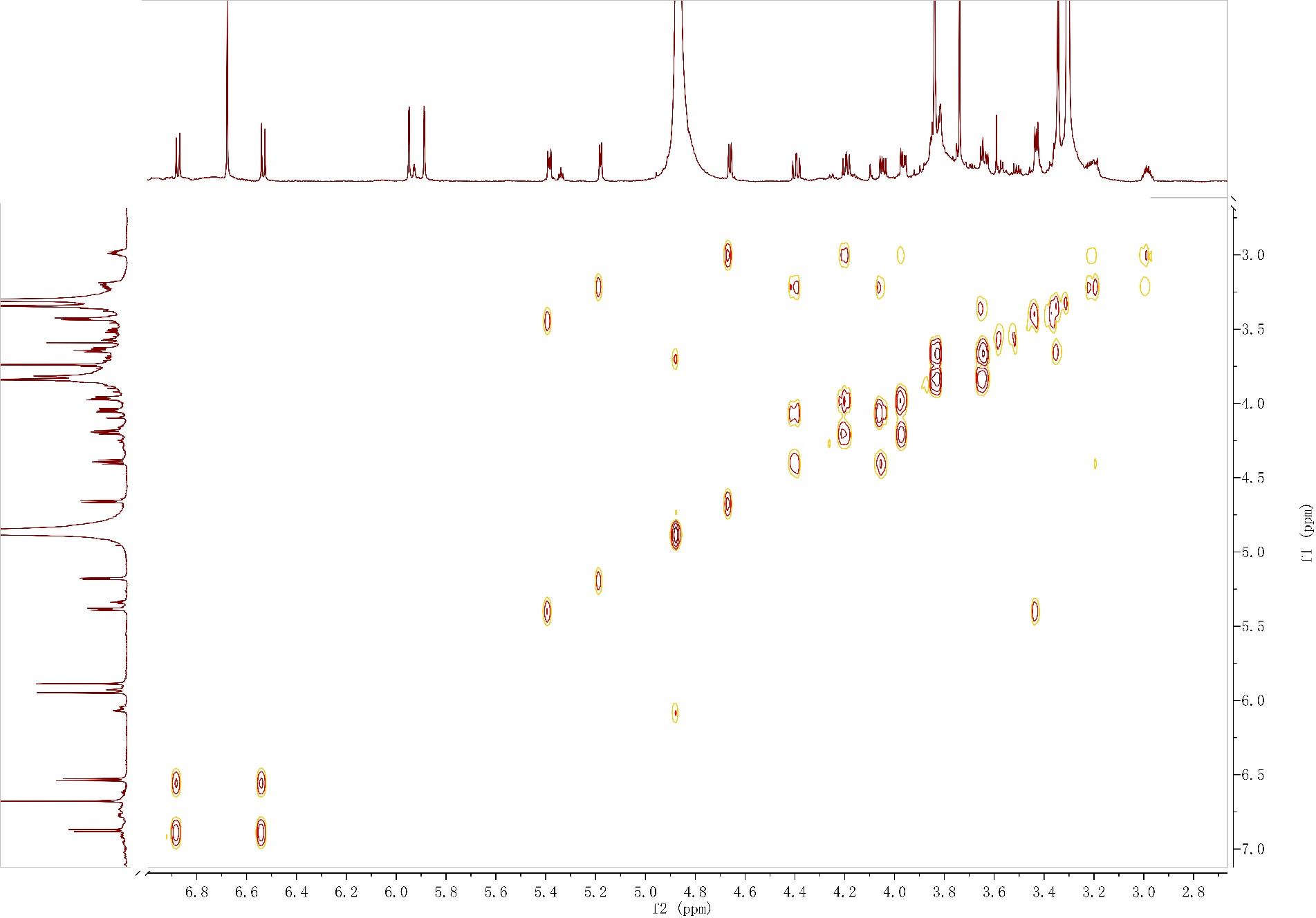


## **Fig. S14** ^1^H-^1^H COSY spectrum of compound **2** in CD_3_OD


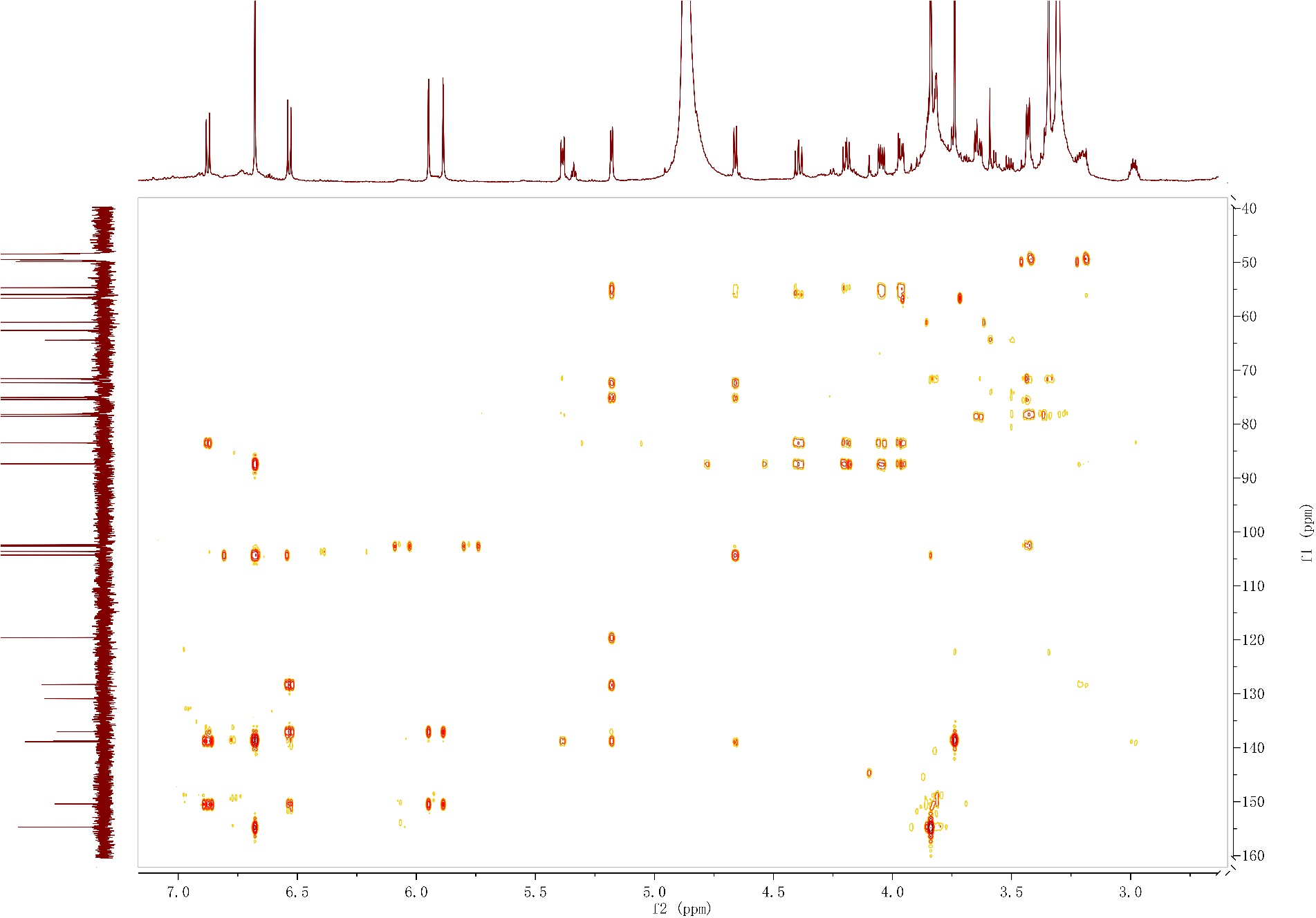


## **Fig. S15** HMBC spectrum of compound **2** in CD_3_OD


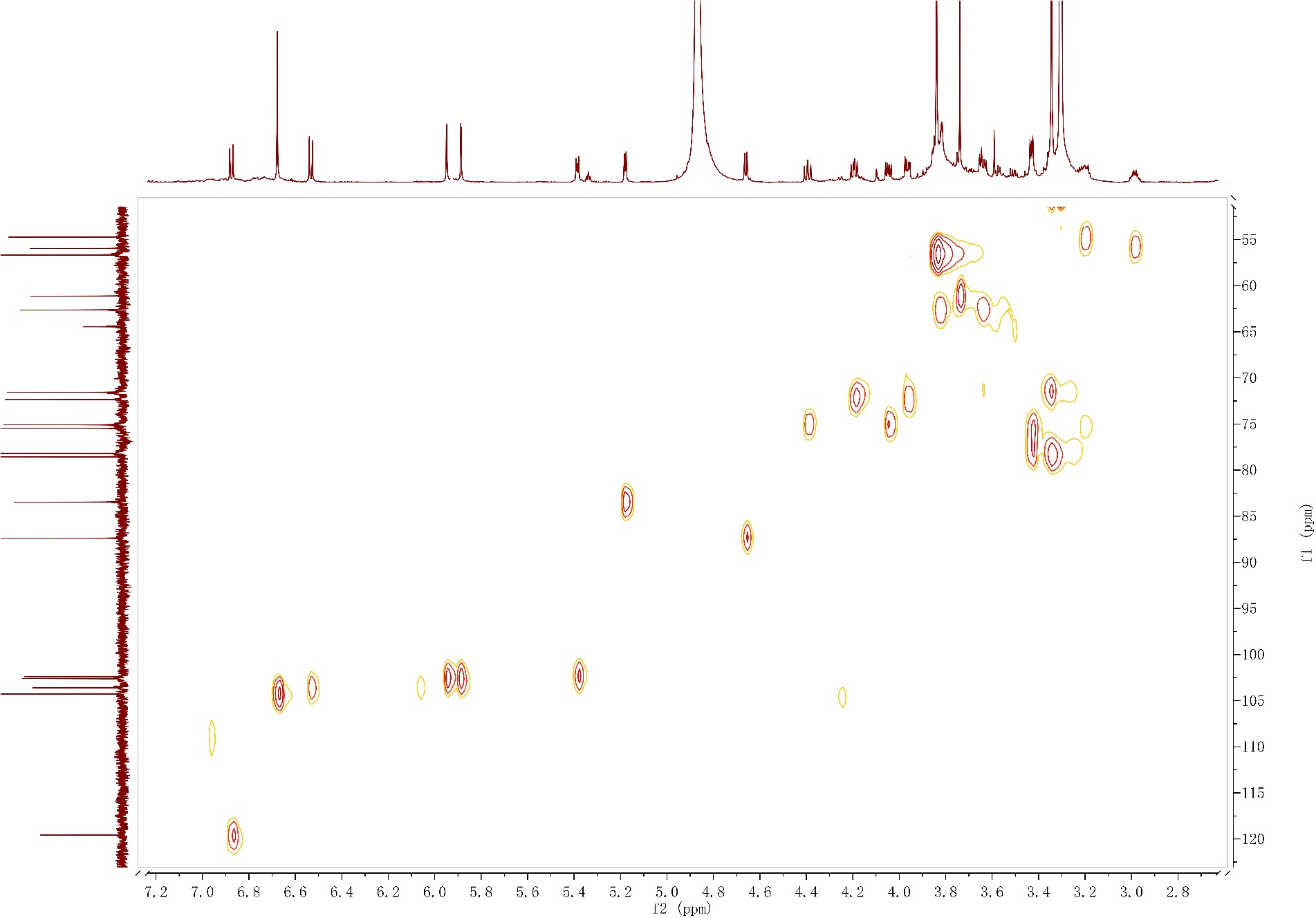


## **Fig. S16** HSQC spectrum of compound **2** in CD_3_OD


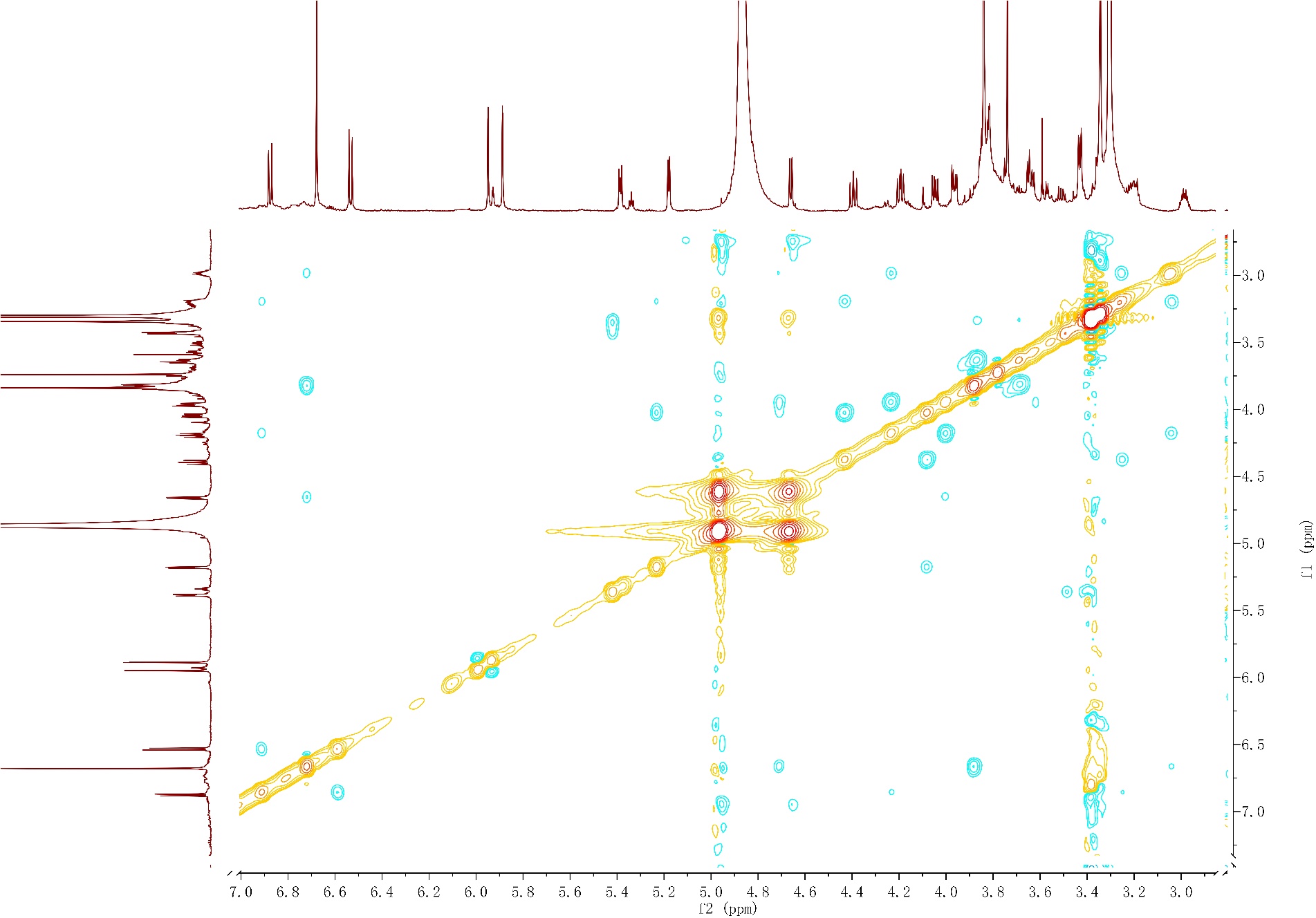


## [**Fig. S17** ROESY spectrum of compound **2** in CD_3_OD](#_Toc61201454)


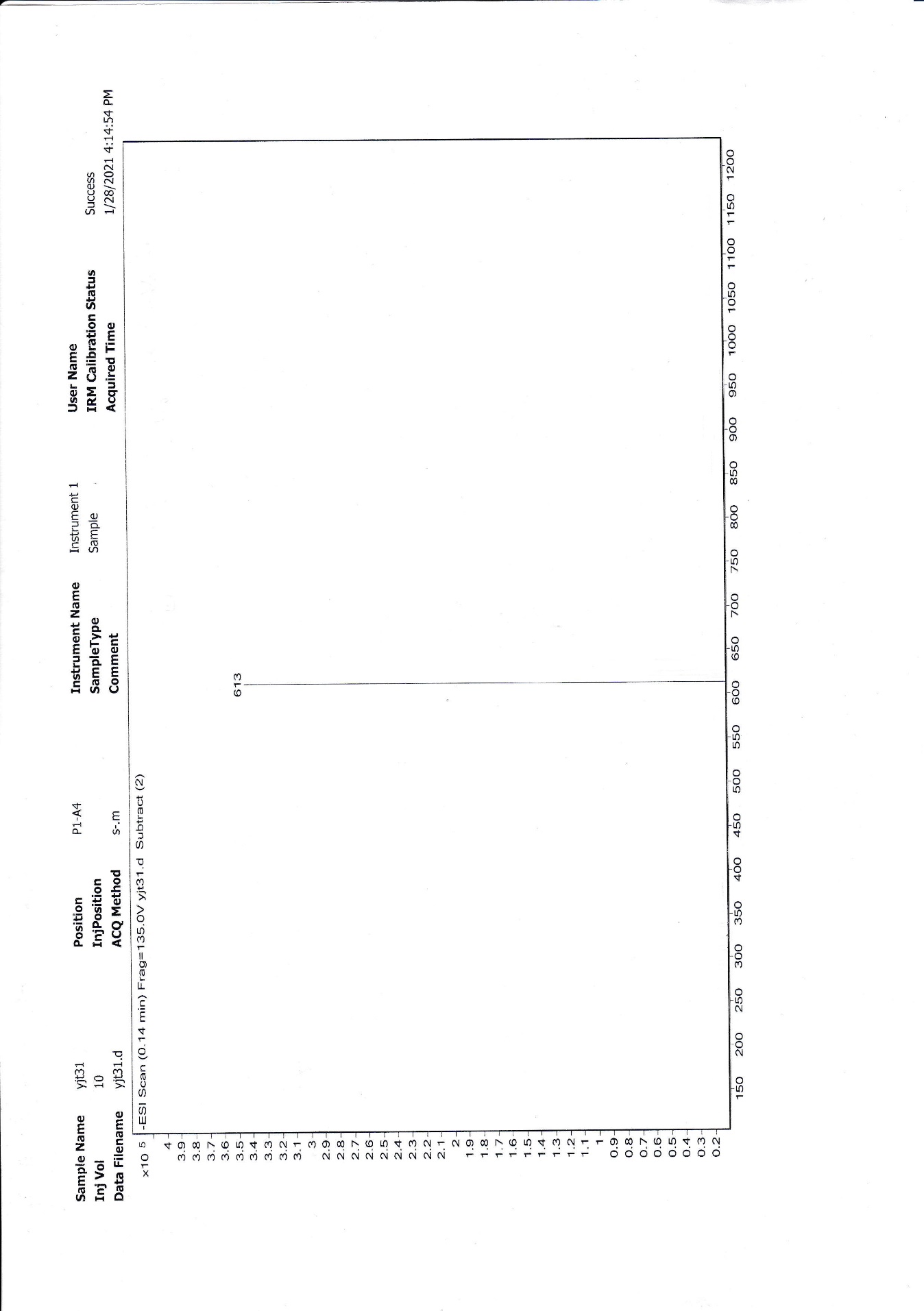


## [[**Fig. S18** The (-)-ESIMS spectroscopic data of compound **2**](#_Toc61201450)](#_Toc61201456)


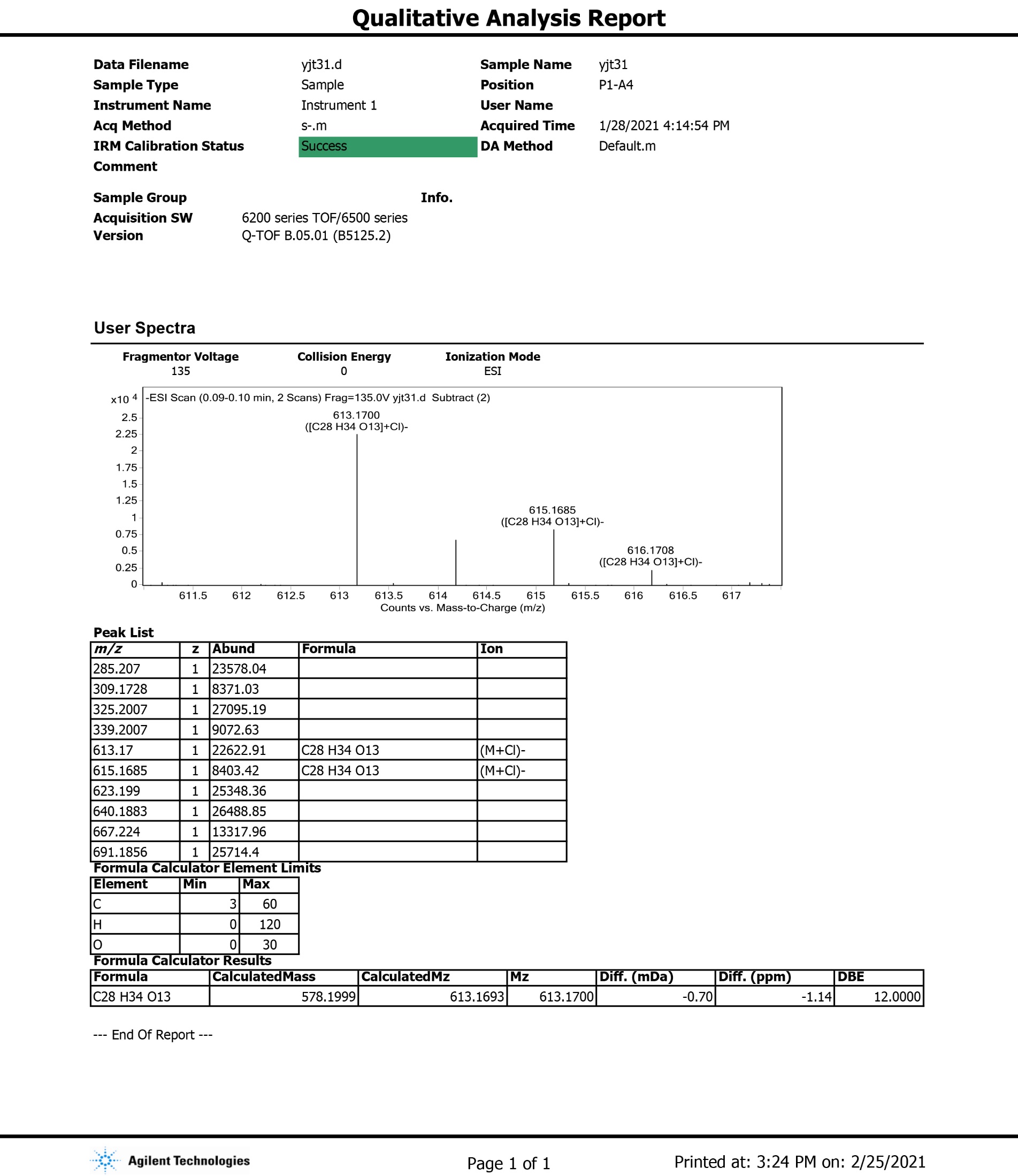


## [[**Fig. S19** The (-)-HRESIMS spectroscopic data of compound **2**](#_Toc61201450)](#_Toc61201456)

## **Fig. S20** The IR spectrum of compound **2**


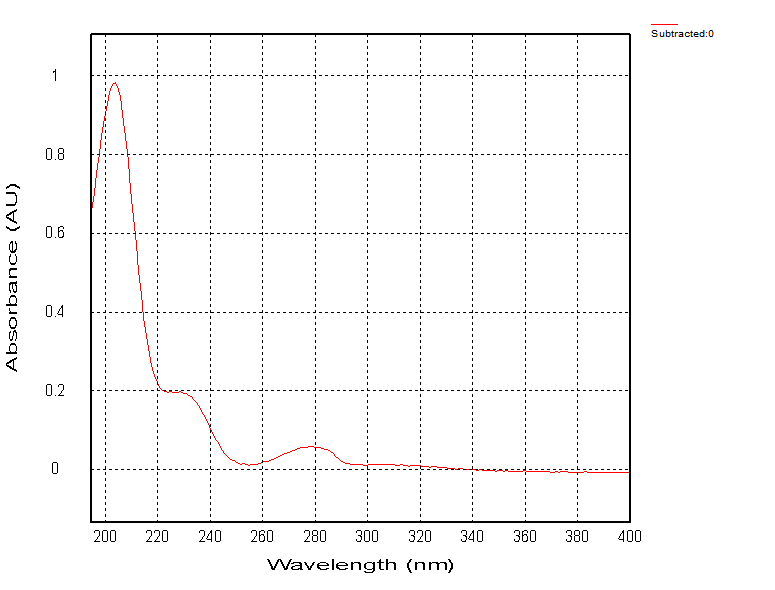


## **Fig. S21** The UV spectrum of compound **2** in CD_3_OD

## **Fig. S22** The ECD spectrum of compound **2** in CD_3_OD
